# Efficacy of a 4-Week Nurse-Led Exercise Rehabilitation Program in Improving the Quality of Life in Women Receiving a Post-Mastectomy Reconstruction Using the Motiva Ergonomix^TM^ Round SilkSurface

**DOI:** 10.3390/ijerph20010016

**Published:** 2022-12-20

**Authors:** Jung Joong Kang, Hyunho Lee, Bom Hui Park, Yu Kwan Song, Soon Eun Park, Robert Kim, Kyung Ah Lee

**Affiliations:** 1 Department of Physical Medicine and Rehabilitation, Booboo Medical Healthcare Hospital, Mokpo 58655, Republic of Korea; 2 Department of Anesthesiology and Pain Medicine, Ulsan University Hospital, University of Ulsan College of Medicine, Ulsan 44033, Republic of Korea; 3 Department of Medical Device Management and Research, SAIHST, Sungkyunkwan University, Seoul 06355, Republic of Korea; 4 Department of Plastic and Reconstructive Surgery, Chung Ju Mirae Hospital, Chungju 27361, Republic of Korea; 5 Department of Medical and Pharmaceutical Affairs, Doctor CONSULT, Seoul 06296, Republic of Korea; 6 Department of Plastic and Reconstructive Surgery, Inje University Haeundae Paik Hospital, 875 Haeun-daero, Busan 48108, Republic of Korea

**Keywords:** breast neoplasms, mastectomy, breast implants, mammaplasty, exercise

## Abstract

We assessed the efficacy of a 4-week nurse-led exercise rehabilitation (ER) program in improving the quality of life (QOL) of breast cancer survivors (BCS) receiving an implant-based breast reconstruction. The eligible patients were equally randomized to either of both groups: the intervention group (*n* = 30; a 4-week nurse-led ER program) and the control group (*n* = 30; a 4-week physical therapist-supervised one). Both after a 4-week ER program and at baseline, the patients were evaluated for the European Organization for Research and Treatment of Cancer Quality of Life Questionnaire Core 30 (EORTC QLQ-C30) and Fatigue Severity Scale (FSS) scores. There was a significantly higher degree of increase in global health status/QOL scores, physical functioning scores, role functioning scores, and emotional functioning scores at 4 weeks from baseline in the intervention group as compared with the control group (*p* = 0.001). However, there was a significantly higher degree of decrease in fatigue scores, nausea/vomiting scores, pain scores, dyspnea scores, and FSS scores in the intervention group as compared with the control group (*p* = 0.001). In conclusion, our results indicate that a 4-week nurse-led ER program might be effective in the QOL in BCS receiving a post-mastectomy implant-based reconstruction using the Motiva Ergonomix^TM^ Round SilkSurface.

## 1. Introduction

Over the past decades, there were advancements in surgical techniques and the prevalent use of breast-conserving surgeries in patients with breast cancer (BC). This led to a dramatic improvement in the prognosis of patients with BC [1]. However, breast cancer survivors (BCS) remain at risk of developing prolonged adverse physical and psychological effects, such as fatigue, vasomotor symptoms, and psychosocial distress [2,3,4]. These adverse effects (AEs) may greatly impair their physical activity (PA), physical performance, and quality of life (QOL) [5,6].

The aerobic and resistance exercise is an important, effective intervention for patients with BC undergoing radiotherapy. It is an easy, cost-effective exercise rehabilitation (ER) program for such patients [7,8]. According to Bekhet AH, et al., aerobic exercise had significant positive effects on cardiorespiratory fitness and non-significant effects on fatigue and weight gain in BCS [9]. Moreover, it was also shown to have a positive effect on improving symptoms of fatigue in BCS [10,11]. Furthermore, it was suggested that it is effective in improving cardiorespiratory fitness, physical function, and muscular strength in BCS [9,11,12,13]. However, this deserves long-term studies. Still, however, there is a paucity of data regarding the efficacy of a nurse-led ER in improving the QOL in BCS receiving a post-mastectomy implant-based reconstruction.

Given the above background, we conducted this study to assess the efficacy of a 4-week nurse-led ER program based on the American College of Sports Medicine (ACSM) exercise guidelines for cancer survivors in BCS receiving a post-mastectomy implant-based reconstruction [14]. To our knowledge, there is a paucity of previous published studies about a nurse-led ER based on the ACSM exercise guidelines in BCS in Korea.

## 2. Theoretical Background

### 2.1. Epidemiology of BC

BC is the most common malignancy in women; according to the International Agency for Research on Cancer (IARC), BC was the second most common cancer worldwide in 2018 and then the most prevalent malignancy in 2020. That is, 2,206,771 patients were newly diagnosed with BC, thus accounting for 11.4% of all cases of malignancies. Moreover, it is the fifth most common cause of death, accounting for 6.9% of all cancer deaths among all the types of malignancies, such as lung, colon, liver, and stomach cancer [15].

In Korea, cancer is the leading cause of death; it has become a major public health concern since 1983. More than 230,000 Korean patients with cancer were identified and it accounted for 1/4 of total death causes in 2017 [16,17]. According to the 2018 Korea Central Cancer Registry (KCCR) data, BC is the most common cancer among women; it accounted for 20.5% of all cases of malignancy in women [15].

### 2.2. Characteristics of BC

BC is a heterogeneous disease on the molecular level. Over the past decades, there was an evolution in treatment concept for it. That is, such evolution is characterized by biologically directed therapies and treatment de-escalation. Despite the inherent molecular heterogeneity, some features that may affect treatment outcomes, such as the impact of locoregional tumor burden or the pattern of metastasis, are commonly observed in patients with BC. Early BC, confined to the breast or only spread to the axillary lymph nodes, is considered a curable disease. Improvements in multimodal therapy raised the rate of treatment success in ≤70–80% of patients with early BC. Based on currently available therapeutic options, however, advanced or metastatic BC is not considered a curable disease. Nevertheless, it can be treated, for which treatment goals are to prolong the survival and to control symptoms with low treatment-associated toxicity. This is essential for maintaining or improving the QOL in patients with advanced or metastatic BC [18].

### 2.3. Cancer-Related Fatigue (CRF)

Fatigue occurs frequently in BCS, thus termed as CRF, and it has a negative impact on their QOL [10]. Its prevalence in breast cancer patients reaches up to 96% [19]. To date, attempts were made to define CRF. It was defined as the “perception of unusual tiredness that varies in pattern and severity and has a negative impact on ability to function in people who have or have had cancer” by the Assessing the Symptoms of Cancer using Patient-Reported Outcomes (ASCPRO) [PROs] working group [20]. In addition, it was also defined as “a persistent subjective sense of tiredness related to cancer or cancer treatment that interferes with usual functioning” by the National Comprehensive Cancer Network (NCCN) practice guidelines for clinical management of fatigue [21]. Furthermore, it was further defined as physical, subjective, temporal, emotional, cognitive, and unusual fatigue that may affect patients’ functions, according to the ASCPRO [20].

It is well known that cancer treatment is closely associated with the CRF and also worsens the existing fatigue [22]. It is also known that advanced cancer patients with a past history of taking chemotherapy are vulnerable to severe CRF [23]. Presumably, this might be because severe fatigue arises from chemotherapy-induced toxicities, such as hematological, gastrointestinal, and neural ones [24].

### 2.4. ER Program for BCS

ER is defined as the restoration and/or maintenance of physical function that is required to perform activities of daily living (ADL) without causing high levels of fatigue or stress. The prescription of ER as a medical treatment is a long-standing notion that gained acceptance in a clinical setting [25]. Numerous randomized controlled trials (RCTs) showed that ER is beneficial for patients with cancer and cancer survivors; it was reported to be effective in improving physical and psychological outcomes [26]. In more detail, ER was shown to reduce many long-term and late unfavorable outcomes of chemotherapy, radiation, surgery, and hormone therapy [27]. Thus, post-treatment ER may be beneficial to cancer survivors. Its clinical benefits in the context of QOL in cancer survivors were well described in the literature. Its indications include cancer survivors who underwent chemotherapy and may be at a risk of developing cardiopulmonary toxicities and aerobic capacity decline and those who did radiation and are at a risk of bone loss and muscular atrophy [28,29,30,31,32].

Both the American Cancer Society (ACS) and the ACSM recommend that BCS receive an aerobic exercise and strength training exercises [14,33,34]. According to a meta-analysis of previous published studies, it was concluded that the ER program has a positive effect on the QOL in BCS [35,36,37]. Previous studies used varying types of outcome measures, such as the timing, type, and duration of interventions, which may complicate a comparison of the results of a meta-analysis [32,35,36]. Moreover, there is a lack of large-scale, prospective, randomized, and long-term controlled trials in this series. It was shown, however, that the ER program, combined with the postoperative adjuvant therapy, was effective in improving the physical fitness and ADLs in BCS [9]. Prospective observational studies showed that it is associated with a reduced risk of cancer recurrence and an improved overall mortality in cancer survivors [33,38,39,40]. This is also accompanied by the report that the ER program improved the physical status and reduced the overall mortality by 24–67% and the breast cancer mortality by 50–53% in women with a diagnosis of BC [41,42,43]. It was also reported that there is an inverse correlation between the PA and co-morbidities in them [44].

It was also reported that the ER program is effective in improving depression and anxiety in BCS receiving adjuvant therapy [12,13,45]. This was seen in patients receiving a moderate physical exercise at a weekly dose of 90–120 min [45].

According to a meta-analysis of trial results about the effectiveness of the ER program in BCS, it had a positive effect on their QOL [9,19,46,47,48,49,50]. This is consistent with recent RCTs showing that it was effective in improving physical, role and social functions, and fatigue [12,13].

### 2.5. Nurse-Led ER Program

Nurses constitute an integral part of health care providers, who perform nursing interventions, such as physical and psychosocial support [51,52,53]. The nurse-led intervention is defined as the intervention where a nurse plays a key role in caring for a patient presenting with a specific episode of a disease; a nurse’s roles include overall coordination, management, and continuity of patient care [54]. From perspectives of family, nurse-led intervention involves family members to support a patient in the management of a disease; it is characterized by self-management of a disease considering that a nurse’s role is within the scope of evidence-based medical practices [54,55,56]. This is because a patient has legal, biological, or emotional relationships with his or her family members [55]. 

According to a previous study about a nurse-led follow-up of women who were treated at a specialist breast care unit in the UK, it was effective in *not only* providing the continuity of care *but also* detecting psychological problems as compared with a physician-led follow-up of them. This suggests that a nurse-led follow-up of BCS might play a key role in meeting their psychosocial needs [57]. Scientific publications about cancer rehabilitation underwent rapid growth. Nurse-led interventions for BC include both exercise/PA and psychoeducation/counseling sessions, and their beneficial effects were recognized. Still, however, nurse-led interventions for cancer rehabilitation are not sufficiently studied. It would therefore be mandatory to accelerate the decision-making process and to establish the best evidence-based practice for multiple stakeholders in BC rehabilitation [58].

### 2.6. QOL in Patients Undergoing Mastectomy

Despite advancements in an understanding of tumor biology *as well as* adjuvant therapies and the trends towards breast-conserving treatments, there was an increase in the use of mastectomy [59]. In the USA, there was an increase in the use of mastectomy from 40% to 51% over the past decade [60]. Moreover, there was a more than 3-fold increase in the number of women undergoing bilateral mastectomies from 9/100,000 in 2005 to 30/100,000 in 2013 [59]. These increases are associated with an increasing demand for contralateral prophylactic mastectomies that are performed through the perception of risk on the part of a patient and in need of optimal symmetry on the part of a surgeon [61,62]. It is known that mastectomy is also the most popular treatment for patients with BC in Korea [63]. Postoperatively, however, these patients may present with physical symptoms, such as pain, lymphedema, a limited range of motion of the shoulder, decreased muscle strength, or sensory changes [64,65]. Such physical symptoms might be decreased during the treatment but they may also remain even postoperatively as factors contributing to impairing both ADL and QOL in patients with BC [66]. Patients with BC might be vulnerable to a feeling of disability due to a loss of breast, distorted body image or self-concept, changes in relationships with family members, including spouse, or fear of recurrence of the disease or death [67]. Patients undergoing mastectomy are vulnerable to poor QOL because of physical and psychosocial alterations. They should therefore be treated with a rehabilitation program to improve physical and psychosocial functions. From this context, diverse types of rehabilitation programs combined with interventions were developed to improve physical, functional, and emotional problems *as well as* QOL in patients with BC [68,69,70].

### 2.7. Implant-Based Reconstruction

Still, the incidence of BC remains fairly constant. Over the past three decades, however, there was a significant increase in the patient survivorship. This eventually made breast reconstruction a reasonable option for the majority of patients undergoing mastectomy [71]. Thus, different types of surgical approaches are required for the post-mastectomy reconstruction of BC; these include implant- or autologous tissue-based reconstruction [72]. The former is known as a minimally-invasive modality, as compared with the latter [73].

Implant-based reconstruction is effective in restoring the natural feel, size, and shape of the breast [74]. It is commonly performed after modified radical mastectomy and nipple- and skin-sparing mastectomy [72,75,76,77]. Depending on differences in the filler or the surface topography of a capsule, diverse types of breast implants are available for implant-based reconstruction [78]. Since the emergence of a silicone gel-filled breast implant in the late 1970s, implant-based reconstruction became the most popular surgical modality for immediate and delayed post-mastectomy breast reconstruction [75]. According to previous cross-sectional studies, patients receiving a silicone gel-filled breast implant achieved relatively higher QOL scores as measured by the BREAST-Q [79,80].

### 2.8. Motiva Ergonomix^TM^ Round SilkSurface

**Figure 1:**
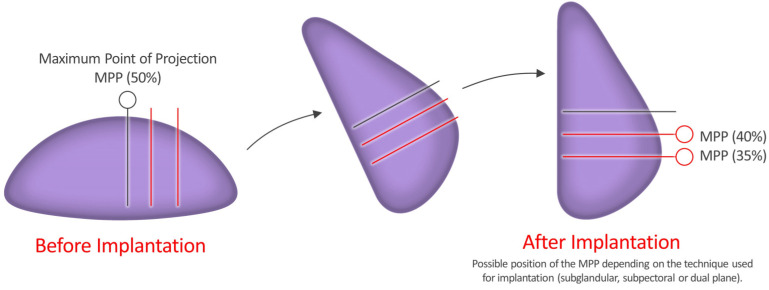
Maximum point of projection (MPP). According to the manufacturer, the Motiva Ergonomix^TM^ Round SilkSurface is advantageous in shifting the MPP to the lower pole of the breast when patients are in a standing posture and to the middle pole of the breast when they lie flat on their back in a similar manner to a natural breast.

The Motiva Ergonomix^TM^ Round SilkSurface (Establishment Labs Holdings Inc., Alajuela, Costa Rica) is equipped with the smallest surface with 49,000 contact points of 16 μm (16,000 nm) depth per cm^2^ [81]. Its properties and characteristics, including the maximum point of projection (MPP), were well documented [82,83,84,85,86]. The MPP is shifted to the lower pole of the breast when patients are in a standing posture, but it moves to the middle pole of the breast when they lie flat on their back. This adjusts the Motiva Ergonomix™ Round SilkSurface to the gravity in a similar manner to a real breast [84] (Figure 1).

## 3. Patients and Methods

### 3.1. Study Patients

The current single-center, prospective, randomized, controlled study was conducted at our medical institution between 16 May and 15 June 2022.

The patients were recruited for the current study according to inclusion/exclusion criteria, as summarized in Table 1.

All the eligible patients submitted a written informed consent. The current study was approved by the Institutional Review Board (IRB) of the Korea National Institute of Bioethics Policy (IRB approval #: P01-202101-19-023); it was conducted in compliance with the relevant ethics guidelines. All the procedures described herein were performed in accordance with the 1964 Declaration of Helsinki and its later amendments or comparable ethical standards.

### 3.2. Rationale of Sample Size Estimation

For the current study, sample size was estimated through the calculation of statistical power. Based on a level of statistical significance (α) of 0.05, a power (1 − β) of 0.80, and an effect size (f) of 0.25, the number of the patients per each group was set at 33 using G*Power software [87]. The rate of loss of follow-up was presumed to be 10%. Therefore, the final sample size for each group was estimated at 36.

### 3.3. Randomization and Compliance

After submitting a written informed consent for study participation at baseline, the eligible patients were given the screening number and they were equally randomized to either of the groups, for which the randomization scheme was generated using the SAS Software Version 9.4 or higher (SAS Institute, Cary, NC, USA) accordingly.

The patients’ compliance was assessed based on a lack of protocol deviation or violation.

**Figure 2:**
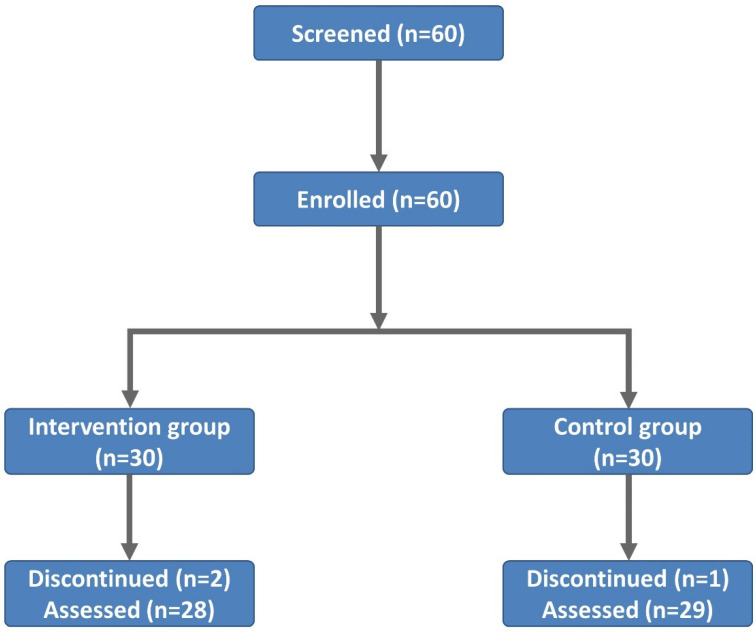
Study flow chart.

A total of 72 patients (*n* = 72) were recruited. Of these, 60 patients (*n* = 60) were eligible for the participation in the current study and then equally randomized to either the intervention group (*n* = 30) or the control group (*n* = 30). However, two patients and one patient of the intervention group and the control group, respectively, dropped out of the current study. They refused to receive an ER program due to their personal situations. Therefore, we finally enrolled a total of 57 patients (*n* = 57) in the current study, 28 and 29 of whom were from the intervention group (*n* = 28) and the control group (*n* = 29), respectively. The study flow chart is shown in Figure 2.

### 3.4. Protocol of Nurse-Led ER Program

The ACSM exercise guidelines recommend that BCS receive ≥three times/week (30 min per session) of moderate aerobic training and an additional two sessions/week of resistance training (8–15 repetitions at 60% of 1-repetition maximum) [14]. The patients of both groups received a 4-week course of a 1hr ER program based on the ACSM guidelines, within the scope of the study protocol, according to which there were no differences in the frequency, intensity, duration, and type of ER between the two groups [34]. Thus, they received a 5-min warm-up, 5-min stretching, a 30-min aerobic exercise (40% of peak oxygen uptake [VO_2_max] followed by a progressive increase in it up to 75%) using a bicycle ergometer (Ergoselect200K, Ergoline, Bitz, Germany), a 15-min resistance exercise (6 different exercises, each of which was repeated 8–12 times) using a latex exercise band (TheraBand^®^, HygenicCorp., Akron, OH, USA), and a 5-min cool-down at a dose of 5 times/week for 60 min/day. We adjusted the tension of a latex exercise band, as previously described [88]. Before receiving a 1-h ER program, the patients of both groups received an exercise stress test (EST). The intensity of exercise was determined based on the maximal heart rate or VO_2_max obtained during an EST [34,88].

The patients of the intervention group received a 4-week nurse-led ER program. However, the patients of the control group did a 4-week physical therapist-supervised ER program. Both the nurse and the physical therapist were female.

### 3.5. Instruments

1. European Organization for Research and Treatment of Cancer Quality of Life Questionnaire Core 30 (EORTC QLQ-C30): The EORTC QLQ-C30 is a 30-item questionnaire integrating system that was developed to assess the health-related QOL in patients with cancer enrolled in a clinical trial [89]. It encompasses five functional scales (physical, role, cognitive, emotional, and social), three symptom scales (fatigue, pain, and nausea and vomiting), a global health and QOL scale and single items about additional symptoms patients with cancer may commonly display (e.g., dyspnea, appetite loss, sleep disturbance, constipation, and diarrhea), as well as the perceived financial impact of the disease and treatment [90]. All the items are scored on a 4-point Likert scale (1 = “Not at all” and 4 = “Very much”). However, two items in the global health/QOL scale are scored on a 7-point linear analog scale [89]. The Korean EORTC QLQ-C30 is the Korean version of the EORTC QLQ-C30, whose reliability and validity were documented. Yun YH, et al. validated the Korean version of the EORTC QLQ-C30. These authors reported that Cronbach’s α coefficients for eight multiple-item scales exceeded 0.70, with the exception of cognitive functioning. Moreover, there were significant correlations between the scales (*p* < 0.01). A multivariate analysis showed that physical and emotional functioning were significant explanatory variables for the global QOL scale (regression coefficients: 0.36, *p* < 0.001; and 0.37, *p* < 0.001; respectively) [91].
2. The Fatigue Severity Scale (FSS): The FSS consists of a total of nine items, each of which is scored on a 7-point Likert scale. There is a positive correlation between the magnitude of FSS scores and the severity of fatigue [92]. It was translated into a Korean language; the reliability and validity of the Korean version of the FSS were documented. That is, Chung KI and Song CH showed that the FSS had a Cronbach’s α coefficient of 0.929 and the Pearson’s correlation coefficient for test–retest reliability was 0.916 (*p* < 0.01) [93].

### 3.6. Patient Evaluation and Criteria

At baseline, the eligible patients completed a questionnaire, provided by the subinvestigator. Then, they were evaluated for the baseline measurements. Data of a questionnaire were collected by an independent research nurse who was blinded to the study details. Items of a questionnaire encompass the demographic and socio-economic characteristics of the patients. These include the age, body mass index (BMI), level of education, marital status, employment status, and monthly household income. The patients’ clinical characteristics were also evaluated; including use of chemotherapy, radiotherapy and hormone therapy, the presence of lymphedema, TNM stage, extent of breast surgery, years after surgery, Eastern Cooperative Oncology Group Performance Status (ECOG PS), and volume of the Motiva Ergonomix^TM^ Round SilkSurface.

Both after a 4-week ER program and at baseline, the patients were evaluated for the EORTC QLQ-C30 and FSS scores [94,95,96]. Changes in the EORTC QLQ-C30 and FSS scores at 4 weeks from baseline served as efficacy outcome measures. All data were entered in the electronic case report form (eCRF).

### 3.7. Statistical Analysis

All data were expressed as mean ± SD (SD: standard deviation). Based on the intention-to-treat (ITT) principle, statistical analysis was conducted using the SPSS version 16.0 (SPSS, Inc., Chicago, IL, USA). We compared differences in changes in the patients’ outcomes at 4 weeks from baseline between the two groups using the Student’s *t*-test. Moreover, we also performed the analysis of covariance (ANCOVA) with adjusted baseline values to explore differences between the two groups. A *p*-value of <0.05 was considered statistically significant.

## 4. Results

### 4.1. Baseline Characteristics of the Patients

All the 57 BCS were included in an ITT analysis. As shown in Table 2, there were no significant differences in the baseline characteristics of the patients between the two groups (*p* > 0.05).

### 4.2. Efficacy Outcomes

**Figure 3:**
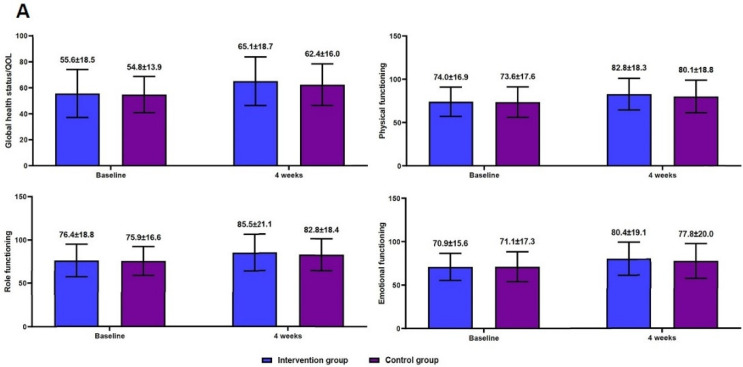

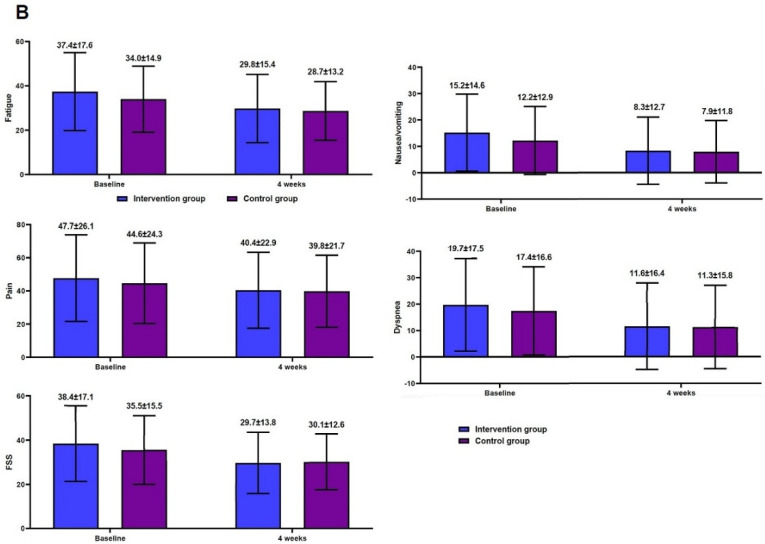
Efficacy outcome measures. Note: QOL, quality of life; FSS, Fatigue Severity Scale. (**A**) An increase at 4 weeks from baseline and (**B**) a decrease at 4 weeks from baseline.

**Figure 4:**
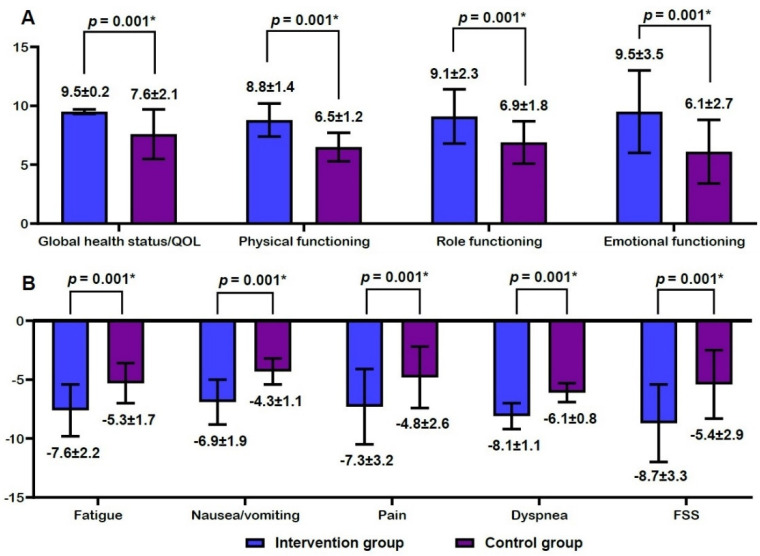
Differences in efficacy outcome measures between the two groups. Note: QOL, quality of life; FSS, Fatigue Severity Scale. (**A**) A higher degree of increase at 4 weeks from baseline and (**B**) a higher degree of decrease at 4 weeks from baseline. * Statistical significance at *p* < 0.05.

Efficacy outcomes are shown in Figure 3 and Figure 4. There were increases in global health status/QOL scores, physical functioning scores, role functioning scores, and emotional functioning scores at 4 weeks from baseline in both groups (Figure 3A). However, there were decreases in fatigue scores, nausea/vomiting scores, pain scores, dyspnea scores, and FSS scores at 4 weeks from baseline in both groups (Figure 3B). Moreover, there was a significantly higher degree of increase in global health status/QOL scores, physical functioning scores, role functioning scores, and emotional functioning scores at 4 weeks from baseline in the intervention group as compared with the control group (*p* = 0.001) (Figure 4A). However, there was a significantly higher degree of decrease in fatigue scores, nausea/vomiting scores, pain scores, dyspnea scores, and FSS scores in the intervention group as compared with the control group (*p* = 0.001) (Figure 4B).

**Figure 5:**
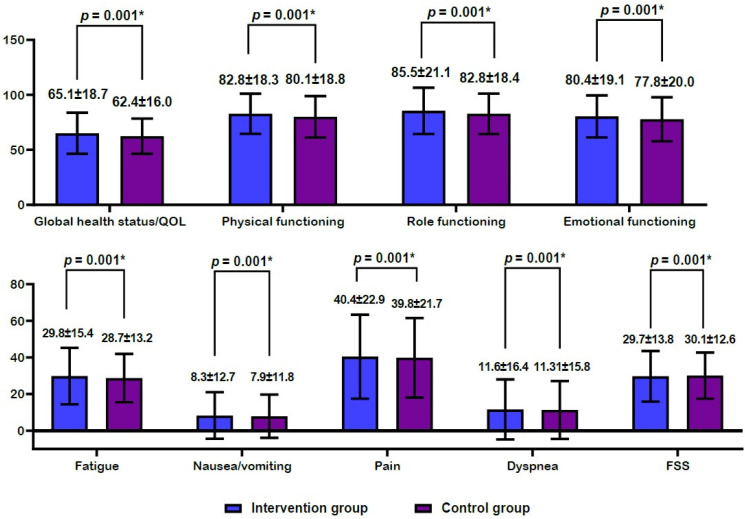
Efficacy outcomes—analysis of covariance. Note: QOL, quality of life; FSS, Fatigue Severity Scale. * Statistical significance at *p* < 0.05.

In the ANCOVA, the global health status/QOL scores, physical functioning scores, role functioning scores, emotional functioning scores, fatigue scores, nausea/vomiting scores, pain scores, and dyspnea scores at 4 weeks were significantly higher in the intervention group as compared with the control group (*p* < 0.05). However, the ANCOVA also showed that the FSS scores at 4 weeks were significantly lower in the intervention group as compared with the control group (*p* < 0.05) (Table 3) (Figure 5).

## 5. Discussion

Surgery is a treatment choice for patients with BC. This has a great psychological impact on their body image, QOL, and sexual life [97]. It remains problematic, however, that patients with BC experience anxiety, worry, stress, fear, depression, and social isolation during the diagnosis and surgical treatment of it. As a result, they are vulnerable to psychological sequelae *as well as* poor treatment outcomes [98,99,100]. 

With advancements in anticancer treatments and early detection of early-stage cancer, the number of BCS in Asia increased, with a 5-year survival rate of ≥90%; the age-standardized 5-year survival of BCS is estimated at 83.1% in China, 89.4% in Japan, and 86.6% in Korea [101]. Both survival and QOL served as important outcome measures in previous peer-reviewed articles about BC survivorship [102,103]. There is a growing interest in understanding of the survivorship experience through PROs [104]. QOL serves as a key measure of PROs, and it is consistently associated with a risk of cancer-related death [105,106,107,108]. It is therefore used to for patient-centered care, clinical decision-making, and health policy or reimbursement decisions [109,110]. BCS are vulnerable to problems with social/emotional support, health habits, spiritual/philosophical views of life, and body image concerns [111,112,113,114]. Their QOL is greatly dependent on their psychosocial condition; it is often impaired during the rehabilitation period immediately after the completion of adjuvant therapy [51,52,115,116]. It may depend on the PA of patients with BC [35,117,118,119,120]. BCS are motivated to make positive changes in their health behaviors. However, they are in need of the ER program to accelerate such changes [121,122].

Exercise is an effective intervention to improve QOL, fitness and physical functioning, to reduce fatigue and to decrease sleep disturbances in both patients with BC and BCS [11,123,124,125,126].

The primary goal of the nurse-led ER program is to provide BCS with psychosocial support, as advocated by a previous literature [127]. It has a positive impact on the physical and psychological well-being of BCS, and it is also effective for stress management [128]. The above psychosocial support is composed of a set of interventions that aim to promote the development of effective coping strategies and to improve the QOL in BCS [129].

Evidence suggests that BCS can achieve improvements in the health status and QOL by actively participating in the nurse-led ER program, for which nurses should help them develop skills, such as disease adaptation *as well as* health promotion [130].

To summarize, our results are as follows: There was a significantly higher degree of increase in global health status/QOL scores, physical functioning scores, role functioning scores, and emotional functioning scores at 4 weeks from baseline in the intervention group as compared with the control group (*p* = 0.001). However, there was a significantly higher degree of decrease in fatigue scores, nausea/vomiting scores, pain scores, dyspnea scores, and FSS scores in the intervention group as compared with the control group (*p* = 0.001).

The above results indicate that a 4-week nurse-led ER program was more effective in improving the QOL as compared with an occupational therapist-supervised one in BCS receiving a post-mastectomy implant-based reconstruction. Our results are in agreement with previous literature advocating the efficacy of ER in improving the QOL in BCS [12,13,14,26,27,28,29,30,31,32,33,34,35,36,37,38,39,40,41,42,43,44,45]. Indeed, RCTs were also conducted to assess the efficacy of a nurse-led ER program for BCS. This showed its efficacy in preventing the occurrence of BC-related lymphedema, restoring the function of the upper limb, and improving the QOL in BCS [131,132,133]. In this regard, a nurse-led ER program and its positive effects on the QOL in BCS deserve special attention. There should be a social consensus on the use of a nurse-led ER for BCS.

A nurse-led survivorship model of care may be a supportive intervention for BCS who are in need of individualized and tailored support and resources that can promote self-management [58]. It would therefore be mandatory for a nurse to have a certain level of scientific fitness literacy, which might be essential for providing scientific ER for diverse types of service objects [134].

However, our results cannot be generalized; there are several limitations of the current study. First, we enrolled a small number of BCS in the current study. Second, we enrolled only the patients receiving the Motiva Ergonomix^TM^ Round SilkSurface for post-mastectomy implant-based reconstruction. The QOL results would be different from the current findings if we enrolled the patients receiving the BellaGel^®^/BellaGel^®^ SmoothFine. According to the news media, the manufacturer, HansBiomed Co. Ltd. (Seoul, Korea), was investigated by the Korean police for using unapproved substances, such as 7-9700 and Q7-4850, and deliberately modifying the shell structure from 5 to 4 layers during the manufacturing process [86,135,136]. Kim JH reported that the manufacturer was previously involved in the Poly Implant Prothèse fraud in Europe [86,135,137]. In 13 November 2020, mandatory recall of the BellaGel^®^ breast implants, including the BellaGel^®^ SmoothFine, was initiated by the Korean Ministry of Food and Drug Safety (KMFDS) [86,135,136,137,138,139]. Later, according to the news dated 3 December 2020, a Korean woman receiving the BellaGel^®^ sustained mental trauma when she became a victim of the first Korean case of a medical device fraud committed by HansBiomed Co. Ltd. in violation of the regulatory requirement enforced by the KMFDS. She complained of severe depressive symptoms, had a handful of hair fall every day, presented with insomnia, and was inclined to commit suicide [140]. The safety of the Motiva Ergonomix^TM^ Round SilkSurface was confirmed in a cohort of Korean women, as previously described [85,86,141]. It was previously reported that the Motiva Ergonomix^TM^ Round SilkSurface might be a device of choice for Korean women who faced a crisis from the first Korean case of a medical device fraud [86]. Nevertheless, this potentially caused a selection bias. Third, we could not completely rule out the possibility that the global QOL scale of the EORTC QLQ-C30 questionnaire has limitations in detecting the negative impacts on health-related QOL, as previously described [142]. It would therefore be challenging to interpret the numerical scores of the EORTC QLQ-C30, although there were improvements in its validation. A large-scale clinical trial can show significant differences in QOL scores. However, controversial opinions exist regarding whether such differences are of clinical relevance [143]. It would therefore be necessary to develop more sensitive patient-reported QOL instruments [144]. Fourth, we failed to use a BC-specific module, the EORTC QLQ-BR23 questionnaire, in measuring the level of QOL in BCS. The EORTC QLQ-BR23 is composed of 23 questions about body image, sexual functioning, sexual enjoyment, future perspective, systemic therapy side effects, breast symptoms, arm symptoms, and hair loss [145]. However, it cannot reflect specific situations of women receiving a post-mastectomy breast reconstruction [145]. Therefore, the EORTC developed the QLQ-BRECON23 to overcome the limitation of the EORTC QLQ-BR23; it is a useful instrument for measuring the QOL in women receiving a post-mastectomy breast reconstruction [146], and its psychometric properties were recently validated by the Korean authors [147]. This deserves further studies.

Nevertheless, our results are of significance in that this is the first report to describe the health-related QOL in a Korean cohort of BCS receiving a post-mastectomy implant-based reconstruction using the Motiva Ergonomix^TM^ Round SilkSurface.

## 6. Conclusions

Based on our results, it can be concluded that a 4-week nurse-led ER program might be effective in QOL in a Korean cohort of BCS receiving a post-mastectomy implant-based reconstruction using the Motiva Ergonomix^TM^ Round SilkSurface. However, further large-scale, multi-center studies are warranted to establish our results.

## Figures and Tables

**Table 1:** Eligibility criteria for the current study.

Inclusion Criteria
(1) Women aged 18 years or older; (2) Women with a confirmed diagnosis of BC according to American Joint Committee on Cancer (AJCC) stages I to III; (3) Women who received an implant-based reconstruction using the Motiva Ergonomix^TM^ Round SilkSurface; (4) Women who postoperatively started adjuvant CTx or RTx; (5) Women who wanted to physically or psychologically tolerate any current and/or future cancer treatments; (6) Women who were in need of the reduction in long-term and late adverse effects of cancer treatment; (7) Women who were in need of the potential delay in any recurrence or progress of the disease.
Exclusion Criteria
(1) Women who concurrently had major health problems that may affect the study participation (e.g., uncontrolled hypertension or other cardiovascular diseases, acute or chronic respiratory diseases); (2) Women with acute arm and shoulder problems for upper body exercises; (3) Women with extreme fatigue, anemia, or ataxia; (4) Women with cognitive dysfunction.

Abbreviations: CTx, chemotherapy; RTx, radiotherapy.

**Table 2:** Baseline characteristics of the patients (*n* = 57).

Variables	Values		*p*-Value
	Intervention Group (*n* = 28)	Control Group (*n* = 29)	
Age (years old)	46.9 ± 8.3	48.1 ± 7.9	0.557
≤39	9 (32.1%)	7 (24.1%)	0.217
40-49	13 (46.4%)	16 (55.2%)	
≥50	7 (21.5%)	6 (20.7%)	
BMI (kg/m^2^)			0.497
<25	13 (46.4%)	17 (58.6%)	
≥25	15 (53.6%)	12 (41.4%)	
Level of education			0.235
≤High school graduates	14 (50.0%)	16 (55.2%)	
≥College or university graduates	14 (50.0%)	13 (44.8%)	
Marital status			0.439
Married	20 (71.4%)	22 (75.9%)	
Unmarried	8 (28.6%)	7 (24.1%)	
Employment status			0.168
Employed	15 (53.6%)	16 (55.2%)	
Unemployed	13 (46.4%)	13 (44.8%)	
Monthly household income			0.063
<USD 924.45	2 (7.1%)	1 (3.5%)	
USD 924.45–1848.89	3 (10.7%)	4 (13.8%)	
USD 1848.89–2773.34	5 (17.9%)	8 (27.6%)	
USD 2773.34–3697.78	10 (35.7%)	9 (31.0%)	
USD 3697.78–4622.23	5 (17.9%)	3 (10.3%)	
>USD 4622.23	3 (10.7%)	4 (13.8%)	
Type of BC			0.398
DCIS	5 (17.9%)	4 (13.8%)	
Non-invasive DCIS	1 (3.6%)	1 (3.5%)	
Invasive DCIS	21 (75.0%)	23 (79.3%)	
Phyllodes tumor	1 (3.6%)	1 (3.5%)	
Use of postoperative CTx	11 (39.3%)	14 (48.3%)	0.529
Use of postoperative RTx	13 (46.4%)	15 (51.7%)	0.716
Use of postoperative HTx	15 (53.6%)	12 (41.4%)	0.426
Presence of lymphedema	22 (78.6%)	19 (65.5%)	0.088
TNM stage			0.653
I	2 (7.1%)	3 (10.3%)	
II	22 (78.6%)	20 (69.0%)	
III	4 (14.3%)	6 (20.7%)	
Extent of breast surgery			0.328
Total mastectomy	3 (10.7%)	7 (24.1%)	
Partial mastectomy	25 (89.3%)	22 (75.9%)	
Years after surgery			0.139
≤1 year	22 (78.6%)	19 (65.5%)	
>1 year	6 (21.4%)	10 (34.5%)	
ECOG PS			0.227
0	5 (17.9%)	6 (20.7%)	
1	18 (64.3%)	19 (65.5%)	
2	3 (10.7%)	3 (10.3%)	
3	2 (7.1%)	1 (3.5%)	
Volume of the Motiva Ergonomix^TM^ Round SilkSurface (cc)			0.077
<250	2 (7.1%)	2 (6.9%)	
250–300	11 (39.3%)	13 (44.8%)	
300–350	9 (32.2%)	8 (27.6%)	
350–400	3 (10.7%)	4 (13.8%)	
≥400	3 (10.7%)	2 (6.9%)	

Abbreviations: BMI, body mass index; BC, breast cancer; DCIS, ductal carcinoma in situ; CTx, chemotherapy; RTx, radiotherapy; HTx, hormone therapy; and ECOG PS, Eastern Cooperative Oncology Group Performance Status. Values are mean ± standard deviation with range or the number of the patients with percentage, where appropriate.

**Table 3:** Efficacy outcomes—analysis of covariance.

Variables	Source	Type Ⅲ Sum of Squares	*df*	Mean Square	F	*p*-Value
Global health status/QOL	Baseline	16,216.118	1	16,216.118	13,210.158	0.001 *
	Group	48.554	1	48.554	39.553	0.001 *
	Error	66.288	54	1.228		
Physical functioning	Baseline	18,624.940	1	18,624.940	1,020,429.109	0.001 *
	Group	73.231	1	73.231	4012.223	0.001 *
	Error	0.986	54	0.018		
Role functioning	Baseline	21,076.493	1	21,076.493	1,022,584.955	0.001 *
	Group	65.107	1	65.107	3158.844	0.001 *
	Error	1.113	54	0.021		
Emotional functioning	Baseline	20,689.941	1	20,689.941	63,536.886	0.001 *
	group	114.141	1	114.141	350.516	0.001 *
	Error	17.584	54	0.326		
Fatigue	Baseline	11,066.604	1	11,066.604	805,790.486	0.001 *
	Group	50.171	1	50.171	3653.072	0.001 *
	Error	0.742	54	0.014		
Nausea/vomiting	Baseline	8109.361	1	8109.361	81,624.965	0.001 *
	Group	72.350	1	72.350	728.237	0.001 *
	Error	5.365	54	0.099		
Pain	Baseline	26,839.665	1	26,839.665	608,759.855	0.001 *
	Group	64.947	1	64.947	1473.081	0.001 *
	Error	2.381	54	0.044		
Dyspnea	Baseline	14,004.433	1	14,004.433	829,104.992	0.001 *
	Group	49.586	1	49.586	2935.641	0.001 *
	Error	0.912	54	0.017		
FSS	Baseline	9418.250	1	9418.250	543,800.299	0.001 *
	Group	106.251	1	106.251	6134.848	0.001 *
	Error	0.935	54	0.017		

Note: *df*, degree of freedom. Abbreviations: EORTC QLQ-C30, European Organization for Research and Treatment of Cancer Quality of Life Questionnaire Core 30; FSS, Fatigue Severity Scale. * Statistical significance at *p* < 0.05.

## Data Availability

The data presented in this study are available on request from the corresponding author. The data are not publicly available due to privacy reasons.
